# Systematic review and meta-analysis of the role of machine learning in predicting postoperative complications following colorectal surgery: how far has machine learning come?

**DOI:** 10.1097/JS9.0000000000003067

**Published:** 2025-07-29

**Authors:** Ali Yasen Mohamedahmed, Shafquat Zaman, Mosaab Agrof, Mohammed A. Adam, Najam Husain, Nuha A. Yassin

**Affiliations:** a Department of General Surgery, University Hospitals Coventry and Warwickshire NHS Trust, Coventry, UK; b Department of Colorectal and General Surgery, University Hospitals of Derby and Burton NHS Trust, Queen’s Hospital Burton, Burton on Trent, UK; c College of Medical and Dental Sciences, School of Medicine, University of Birmingham, Birmingham, UK; d Research Department, Kuwait University, Kuwait City, Kuwait; e Global Surgery Department, Harvard Medical School, Boston, MA, USA; f Department of Colorectal and General Surgery, University Hospitals Birmingham NHS Trust, Birmingham, West Midlands, UK

**Keywords:** colorectal surgery, machine learning, postoperative complications, predictive modeling, systematic review and meta-analysis

## Abstract

**Background:**

To systematically evaluate the clinical utility of machine learning in predicting postoperative outcomes following colorectal surgery.

**Methods:**

A systematic literature search was conducted using PubMed, MEDLINE, Embase, and Google Scholar. Clinical studies investigating the role of machine learning models in predicting postoperative complications following colorectal surgery were included. Outcome measure was area under the curve for the model under investigation. The area under the curve and standard error were pooled using a random effects model to estimate the overall effect size. Statistical analyses were performed using the MedCalc (version 23) software, and the results presented as forest plots.

**Results:**

Eighteen eligible articles were included. These reported outcomes on postoperative complications, namely anastomotic leak, mortality, prolonged length of hospitalization, re-admission rates, risk of bleeding, paralytic ileus occurrence, and surgical site infection. Pooled area under the curve for anastomotic leak was 0.813 [standard error: 0.031, 95% confidence interval (0.753–0.873)]; mortality 0.867 [standard error: 0.015, 95% confidence interval (0.838–0.896)]; prolonged length of stay 0.810 [standard error: 0.042, 95% confidence interval (0.728–0.892)]; and surgical site infection 0.802 [standard error: 0.031, 95% confidence interval (0.742–0.862)], respectively.

**Conclusion:**

Machine learning methods and techniques are displaying promising clinical utility and applicability in accurately predicting the risk of developing complications following colorectal surgery. Future well-designed, adequately powered, multi-center studies are needed to investigate the usefulness and generalizability of these novel approaches in optimizing peri-operative surgical care.

## Introduction

Colorectal resections are performed for a wide variety of conditions, including malignancies, inflammatory bowel disease (IBD), diverticular disease, volvulus, polyposis syndromes, infections, bowel obstruction, or following trauma. Colorectal cancer is the third most common malignancy worldwide[1] and the second leading cause of cancer-related mortality[2]. Moreover, a significant proportion of patients with IBD (ulcerative colitis and Crohn’s disease) require surgery either for symptom control (disease refractory to medical therapy) or as a consequence of developing a complication. The type of surgery is dependent upon disease location and mapping, and may involve the small bowel, colonic segmental resection, and surgery to the perineum.

Resectional colorectal surgery performed for both benign and malignant disease can potentially be curative but is associated with significant morbidity and mortality. Postoperative complications following cancer surgery can impact overall and disease-free survival[3] and may have an adverse impact on quality of life. These include anastomotic leaks (ALs), surgical site infections (SSIs), intra-abdominal abscess formation, and hemorrhage.

Postoperative complications vary in severity, and early recognition and treatment are critical in achieving a favorable outcome[3]. Despite advances in surgical techniques, the reported AL rate varies between 2.8% and 30%[4], with these differences being associated with non-standardized definitions and grading systems used to define ALs[5] and SSIs estimated at 2.4%-21.6%[5]. The occurrence of such complications can negatively impact patient well-being and health care providers by increasing length of stay (LOS), readmission rates, cancellation of elective lists, and significant cost implications.

Machine learning [branch of artificial intelligence (AI)] is concerned with the study and use of statistical algorithms derived from data and used to generate predictions[6]. Inferences can be drawn from patterns in data either through supervised or unsupervised learning. The application of machine learning techniques in various fields, including medicine, has been gaining popularity in an attempt to predict patient outcomes accurately[7].

A number of predictive tools and risk stratification models are already in use to stratify an individual’s risk of postoperative morbidity and mortality[8] and are important in aiding peri-operative decision-making, especially in major surgical procedures. These include the American Society of Anaesthesiologists (ASA) score, the American College of Surgeons Surgical Risk Calculator[9], and the Physiologic and Operative Severity Score for the Enumeration of Mortality and Morbidity (POSSUM)[10] score.

However, machine learning models may be more comprehensive and superior to existing linear risk prediction tools. This study aims to systematically evaluate the clinical utility of machine learning in predicting postoperative outcomes following colorectal surgery.

## Methods

The systematic review and meta-analysis were designed, performed, and reported according to the following guidelines: Cochrane Handbook for Systematic Reviews of Interventions[11]; Preferred Reporting Items for Systematic Reviews and Meta-Analyses (PRISMA)[12]; Assessing the Methodological Quality of Systematic Reviews (AMSTAR 2)[13]; and Transparency In The reporting of Artificial INtelligence (TITAN 2025)[14]. The TITAN checklist is provided in Supplemental Digital Content, Appendix 1, available at: http://links.lww.com/JS9/E785.

The protocol of this review was registered at the International Prospective Register of Systematic Reviews (registration number: CRD42025638998 available at: https://www.crd.york.ac.uk/prospero).

### Search strategy

Studies reporting machine learning models used to predict postoperative complications following elective or emergency colorectal surgery (performed either through an open or minimally invasive approach for benign and malignant pathologies including restoration of bowel continuity) were deemed eligible for inclusion. The types of procedures included were as follows: ileo-caecal resections; right (extended) hemicolectomies; left hemicolectomies; subtotal colectomies; segmental colonic resections; sigmoid colectomies; anterior (low) resections; pan-proctocolectomies; abdominoperineal resections; proctectomies; Hartmann’s resection/reversal; diverting stoma; intestinal bypass; and insertion of self-expanding metallic stents.

Literature search was performed using the following online electronic databases: PubMed, MEDLINE, Embase, and Cochrane Central Register of Controlled Trials (CENTRAL) up to and including the 20/01/2025. Additionally, the reference list of relevant studies were reviewed manually to identify further eligible studies. A combination of the following search terms were used: “machine learning,” “artificial intelligence,” “AI,” “postoperative complication,” “colorectal surgery,” “gastrointestinal surgery,” and “colectomy.”

**HIGHLIGHTS**

- Role and potential clinical utility of machine learning techniques in predicting postoperative complications following colorectal surgery.
- Overall pooled area under the curve for anastomotic leak was 0.813; mortality 0.867; increased length of hospital stay 0.810; and surgical site infection 0.802.
- Machine learning pipelines display promising clinical utility and good predictive ability in determining postoperative complications following colorectal resectional surgery, and should be further investigated with better design standardization and reporting of results.

Supplemental Digital Content, Appendix 2, available at: http://links.lww.com/JS9/E786 shows the search strategy used in the literature search.

### Eligibility and study selection criteria

Inclusion criteria were all clinical studies using machine learning tools to predict postoperative complications in patients undergoing colorectal surgery. No restrictions were applied to the indication for surgery, and both benign and malignant pathology were considered. Furthermore, all types of colectomies, ileocecal resection and proctectomy were deemed eligible. Study exclusion criteria were as follows: anorectal conditions (including common perineal approaches such as Delorme’s and Altemeier’s operation) and appendicectomy, non-clinical articles, expert and technical opinions, conference abstracts, and letters to the editor.

Titles and abstracts of selected articles were screened independently by two authors, and the full text of potentially eligible articles were retrieved and reviewed. Disagreements were resolved through consensus following discussion with the authorship team.

### Data extraction and outcomes

Two authors extracted data independently, which were revised by a third author using a standard Microsoft Excel spreadsheet after pilot-testing. The information collected from each study included name of first author, year of publication, country of origin, total number of patients, inclusion and exclusion criteria, assessed postoperative complication(s), machine learning model and training method, area under the curve (AUC) for the assessed complication/outcome and important predictors/variables. In instances where more than one machine learning model was utilized, the model with the greatest AUC was reported.

### Risk of bias assessment

The prediction model Risk of Bias Assessment Tool (PROBAST) was used to evaluate the risk of bias (ROB)[15]. The PROBAST tool, informed by a Delphi procedure and refined through piloting, evaluates ROB and applicability. ROB comprises four domains: participants, predictors, outcome, and analysis. These domains, containing 20 questions, help facilitate a structured judgement of ROB in a study that develops, validates, or updates a prediction model. Moreover, the applicability assessment covered three domains: Participants, Predictors, and Outcomes. PROBAST enables a transparent and focused approach to identify potential flaws in study design, conduct, and data analysis, resulting in distorted estimates of a particular model’s predictive performance.

### Data synthesis and statistical analyses

The meta-analysis was performed using the MedCalc (version 23) software, and the results were presented as forest plots. The AUC, along with the standard error (SE), was pooled from included studies using a random effects model to estimate the overall effect size and pooled AUC with a 95% confidence interval (CI). Where SE was not reported, the 95% CI was used instead to estimate the SE[16]. Heterogeneity was evaluated using the Cochran Q test and I^2^ statistic. An I^2^ value exceeding 50% signified significant levels of between-study heterogeneity, and a value of 0% indicated no heterogeneity.

To check for possible sources of heterogeneity and evaluate the robustness of our results, sensitivity analysis was performed by calculating the fixed effects model.

## Results

Our initial search identified a total of 421 studies. Following review of titles and abstracts, 275 were excluded due to duplication or lack of relevance. This resulted in 146 studies remaining for full-text screening, and 128 were excluded for not meeting the inclusion criteria. A total of 18 studies^[17–34]^ were included in the final systematic review.

**Figure 1.**
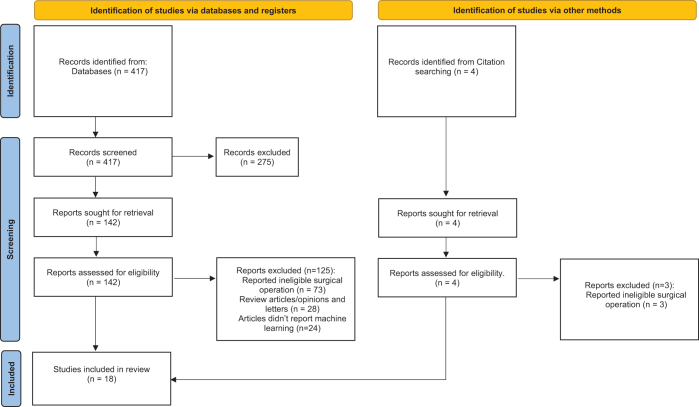
PRISMA flow chart.

Figure 1 demonstrates the PRISMA flow chart.

The characteristics of the included studies are presented in Table 1, and a summary of the machine learning models’ performance is provided as Supplemental Digital Content, Appendix 3, available at: http://links.lww.com/JS9/E787.

**Table 1:** Characteristics of included studies

Study	Country	Population and Inclusion Criteria	Machine Learning Model	AUC (best model)	Important Variables
Adams 2013[17]	UK	76 patients (January 2005—December 2011)	ANN	0.89	Anastomosis location, de-functioning stoma, elective/emergency operation, heart rate, systolic blood pressure, temperature, white cell count, neutrophil count, platelet count, albumin level.
		**Outcome**: AL.	**Input variables**: 61		
			Data split to training and validation (3:1 ratio)		
Francis 2015[18]	UK	275 consecutive patients (2002–2009)	ANN	Delayed discharge: 0.82	Operative duration, blood loss, stoma formation, discontinuation IV fluids on POD1, epidural failure, and failure to mobilize POD1, ileus and catheterization.
		**Outcomes**: delayed discharge and re-admission.	Data split into 182 patients for training and 81 for validation.	Re-admission 0.68	
		**Inclusion criteria**: laparoscopic colorectal resections (right, left, or subtotal colectomies, segmental resections, sigmoid colectomy and rectal resections) for malignant pathologies.	Re-admission rate 12.5% and delayed discharge (LOS >7 days) rate 24.4%.		
Chen 2018[19]	USA	12,402 patients	LR and XGB	XGB: 0.819	Anemia, hemophilia, nutrition, surgical length, weakness, mobility, heart failure, activity level
		**Outcome**: bleeding within 7-days postoperatively.	**Input variables**: 117 variables	LR: 0.773	
		**Inclusion criteria**: all colorectal procedures.	Bleeding rate 12.5%.		
			10 runs of 10-fold cross-validation.		
Azimi 2019[20]	Georgia	308 patients (2016–2017)	Multiple machine learning models.	0.89	Age, serum sodium concentration, BUN concentration, hematocrit, platelet count, surgical procedure time, and LOS.
		**Outcome**: all postoperative infections.	**Input variables**: 38 variables.		
		**Inclusion criteria**: colorectal resections.	Data are split into 52% for training and 48% for validation.		
			Postoperative infection rate 8.7%.		
Bosch 2021[21]	Netherlands	62,501 patients (2011–2016)	Multivariable logistic regression, elastic	Mortality: 0.82	Hypertension, myocardial infarction, chronic obstructive pulmonary disease, and asthma
		**Outcomes**: 30-day mortality, re-admission.	Net regression (best result), SVM, RF, and XGB.		
		**Inclusion criteria**: all patients undergoing colorectal cancer surgery during the study period.	**Input variables**: 117 variables.	Re-admission: 0.63	
			Data set split chronologically into a test set (19%, 2016) and a training set containing 81% (2011–2015).		
			Mortality rate 2.7%.		
			5-fold cross-validation on the training set using stratified splitting.		
Degett 2021[22]	Denmark	1901 patients (2014–2017)	LR	0.80	Age, performance status, alcohol, smoking and primary surgical procedure.
		**Outcome**: 90-day mortality rate.	**Input variables**: 10 variables.		
		**Inclusion criteria**: emergency surgical procedure for colorectal cancer.	Data split into training (2014–2016, 1450 patients) and validation (2016-2017, 451 patients).		
		**Exclusion criteria**: if registered with an elective surgical procedure before the acute procedure, abdominoperineal excision, if deceased, migrated, or lost to follow-up before the date of surgery.	Mortality rate 19%.		
Mazaki 2021[23]	Japan	256 patients (2017–2021)	ANN	0.77	Age, gender, BMI, ASA score, preoperative hemoglobin, emergency procedure, operative time, hemorrhage, anastomosis level, circular stapler, diverting stoma, pathological T-status, pathological N-status, lymphatic invasion negative, venous invasion, histological type, postoperative complication
		**Outcome**: AL	**Input variables**: 18 variables.		
		**Inclusion criteria**: patients underwent curative surgery for left-sided colorectal surgery with anastomosis.	165 patients for training and 100 patients for validation.		
		**Exclusion criteria**: anastomosis <5 cm from the anal verge and preoperative therapy.			
Wen 2021[24]	USA	5,220 patients (underwent AR for rectal cancer from 2009 to 2019)	Random forest	0.85	Distance of tumor to the anal verge, nCRT, diabetes, surgeon volume, gender, stenosis or obstruction, preoperative hemoglobin, surgical approach.
		**Outcome:** AL	**Input variables**: 20 variables.		
		**Inclusion criteria**: rectal cancer patients underwent AR with TME; complete clinical data.	Data split into 80% training set and 20% validation set.		
			AL rate 6.2%.		
		**Exclusion criteria**: local excision, Hartmann surgery; tumor >15cms from the anal verge; multiple primary colorectal carcinomas.			
Bräuner 2022[25]	Denmark	65,612 patients (2001–2019)	LASSO (best model), decision tree, RF, XGB, K-nearest neighbor, MLP, and AdaBoost models.	30-day: 0.87	Age group, ASA, primary malignant neoplasm of the splenic flexure of the colon, treatment category, emergency operation, exploratory surgery, exploratory laparotomy, colonic stent, and ileocolic resection.
		**Outcomes**: 30, 90, 180-day mortality.	**Input variables**: 38 variables.	90-day: 0.87	
		**Inclusion criteria:** > 18 years old and underwent colorectal cancer surgery.	Data randomly split into a training set (75%) and test set (25%).		
			Mortality rates at 30, 90, and 180 days—5.42%, 8.53%, and 11.42%, respectively.	180-day: 0.88	De-functioning stoma, perforation.
Lin 2022[26]	Denmark	23,907 patients (2014–2019)	LASSO (best result), logistic regression, XGB, RF, K nearest neighbor, multilayer perceptrons and decision trees.	AL: 0.6	Sex, age group, BMI, ASA, alcohol consumption, Charlson Comorbidity score, performance status, smoking status, Family history
		**Outcomes**: Clavien–Dindo grade ≥3B complications and AL.	**Input variables**: 16 variables.	CD: ≥ 3B 0.70	
		**Inclusion criteria:** > 18 years of age, any resection for colorectal cancer.	Data split randomly into training (75%) and validation (25%) set.		
			AL rate 5.4%, and CD grade ≥ 3B complications 12.4%.		
			Three-fold cross-validation strategy for hyperparameter optimization.		
Chen 2023[27]	USA	275,152 patients (2012–2019)	RF, XGB, and DNN.	DNN: 0.76	Organ space SSI present at the time of surgery, operative time, antibiotics bowel preparation, surgical approach, procedure, BMI, wound classification, age, weight, surgery indication.
		**Outcome**: SSI.	**Input variables**: 49 variables.		
		**Inclusion criteria**: colectomy and proctectomy from the American College of Surgeons National Surgical Quality Improvement Program (NSQIP) database	Data split into training and validation (2012 to 2018, 80% and 20%), and 2019 data used for the test set.		
			SSI rate 10.7%.		
			5-fold cross-validation.		
Shen 2023[28]	China	833 patients (underwent LAR 2008–2019)	LASSO and SLM	LASSO: 0.79	Hypertension, cT4 stage, intra-operative blood loss >100 mL, operative time >160 min, tumor size, and tumor location.
		**Outcome**: AL	**Input variables**: 19 variables.	SLM: 0.75	
		**Inclusion criteria**: mid-low rectal cancer, LAR with curative intention without a diverting stoma.	Data were split into 70% for training and 30% for validation.		
		**Exclusion criteria**: high rectal cancer, synchronous distant metastasis, emergent surgery.	AL rate 6.2%		
			10-fold cross-validation		
Agnes 2024[29]	USA	941 patients (2016–2021)	ANN	AL: 0.84	Baseline and POD 1 to 3 creatinine, hemoglobin, neutrophil count, lymphocyte count, monocyte count, white blood cell count, platelet count, C-reactive protein level.
		**Outcomes**: AL and re-admission.	Fifty tours were used for the model training, and no boosting was used.	Re-admission: 0.95	
		**Inclusion criteria:** > 18 years, elective colorectal surgery for primary colorectal cancer.	AL rate 3.1%; re-admissions 6.8%.		
		**Exclusion criteria**: multi-visceral resection (including exenteration or proctocolectomy), beyond-TME resection or metastasectomy, no anastomosis, less than 90 days postoperative follow-up.			
Brydges 2024[30]	USA	316 patients (2020–2021)	8 machine learning models	0.84	Age, BMI, gender, kidney disease, anemia, arrhythmia.
		**Outcome**: postoperative ileus.	**Input variables**: 29 variables.		
		**Inclusion criteria**: adult patients who underwent colorectal surgery.	Data were split into 60% for training, 20% for validation and 20% for test.		
		**Exclusion criteria**: multi-visceral resections, re-operations or combined primary and metastatic resections, no follow-up within 90 days after surgery.	Ileus rate 6.3%.		
			Five-K-fold cross-validation		
Li 2024[31]	China	322 patients (2018–2021)	Random forest, support vector machine, and DNN.	DNN: 0.87	ASA, operation time, albumin, sutures used at closure, and surgical approach.
		**Outcome**: SSI.	**Input variables**: 16 variables.		
		**Inclusion criteria**: patients who underwent right hemicolectomy and postoperative pathological diagnosis of colon cancer.	Data were split into training (70%) and test (30%).		
		**Exclusion criteria**: previously diagnosed colon cancer, prior treatment history, incomplete medical records, and patients undergoing emergency surgery for other conditions.	SSI rate 4.3%.		
Nwaiwu 2024[32]	USA	14,935 patients (2003–2017)	Decision tree, RF, and ANN (best result).	AL: (open/Lap): 0.88/0.93	Diagnosis-related group, insurance type, gender, race.
		**Outcomes**: AL, prolonged LOS, inpatient mortality.		Prolonged LOS: (open/Lap):	
		**Inclusion criteria**: Adults with colonic neoplasia, elective laparoscopic and open partial colectomy with anastomosis, no diverting stoma.	Inpatient mortality 1.03%, prolonged LOS 10.8%, and AL 0.99%.	0.84/0.88	
		**Exclusion criteria**: Rectal neoplasia, lack of information on one or more demographics of interest.	A 5-fold cross-validation.	Mortality: (open/Lap): 0.90/0.92	
Taha-Mehlitz 2024[33]	Switzerland	1244 patients (2012–2020)	Random forest.	0.78	Surgical procedure, emergency surgery, renal function, indication, liver metastasis, leukocytosis, preoperative steroid use, technique (hand-sewn), active smoking, prior abdominal surgery.
		**Outcome**: AL	**Input variables**: 21 variables.		
		**Inclusion criteria**: patients underwent colectomy and anastomosis and follow-up of at least 6 months.	Data were split randomly into a test set (10%) and a train set (90%).		
		**Exclusion criteria**: no follow-up, colonic resection without anastomosis, death after surgery, or a diverting stoma still in place at last follow-up.	A five-fold cross-validation methodology was employed from model training.		
Traeger 2024[34]	Australia	504 patients.	Multivariate logistic regression, decision trees, radial basis function and MLP.	MLP: 0.80	Postoperative hypokalemia, surgical approach, and opioid use, sarcopenia.
		**Outcome**: postoperative ileus.	**Input variables**: 30 variables.		
		**Inclusion criteria**: aged over 18 years, underwent open or laparoscopic major large bowel surgery.	Data were split randomly into 80% for training and 20% for testing.		
		**Exclusion criteria**: emergency surgery, small bowel resections and pelvic exenterations.	Ileus rate 36%.		

The best-performing model was included when multiple machine-learning models were used. Important variables are ranked by the model as being significant in predicting the outcome of interest.

AUC: area under the curve, AL, anastomotic leak, ANN: artificial neural network, ASA: American Society of Anaesthesiologists, BMI: Body Mass Index, IV: intravenous, LASSO: Least Absolute Shrinkage and Selection Operator, SLM: small language model, MLP: multilayer perceptron, DNN: deep neuronal network, XGB: gradient boosting, RF: random forest, SVM: support vector machine, LR: logistic regression, POD: postoperative day, SSI: surgical site infection, LOS: length of stay, Lap: laparoscopic, BUN: blood urea nitrogen, CD: Clavien Dindo, AR: anterior resection, LAR: low anterior resection, TME: total mesorectal excision, nCRT: neoadjuvant chemoradiotherapy

## Risk of bias and applicability assessment

Overall, most of the included studies demonstrated a low ROB across all domains. In the participants’ domain, 14 studies (78%) showed a low ROB, whilst four studies (22%) presented an unclear ROB. Only one study showed a high ROB in the predictors’ domain, with two studies showing an unclear ROB. For the analysis domain, one study (6%) had a high ROB, and four studies (22%) revealed an unclear ROB. Overall, ROB assessment showed 11 (61%) of the included studies as being low risk, five (28%) unclear risk, and two (11%) as high risk.

For the applicability assessment, in the participants and predictors domains, three studies (17%) and one study (6%) had high concerns, respectively. Moreover, three studies (17%) had some concerns, and one (6%) had high concerns in the outcome domain. Overall, applicability assessment indicated that 13 studies (72%) were of low concern, four (22%) high concern, and one (6%) unclear.

**Figure 2.**
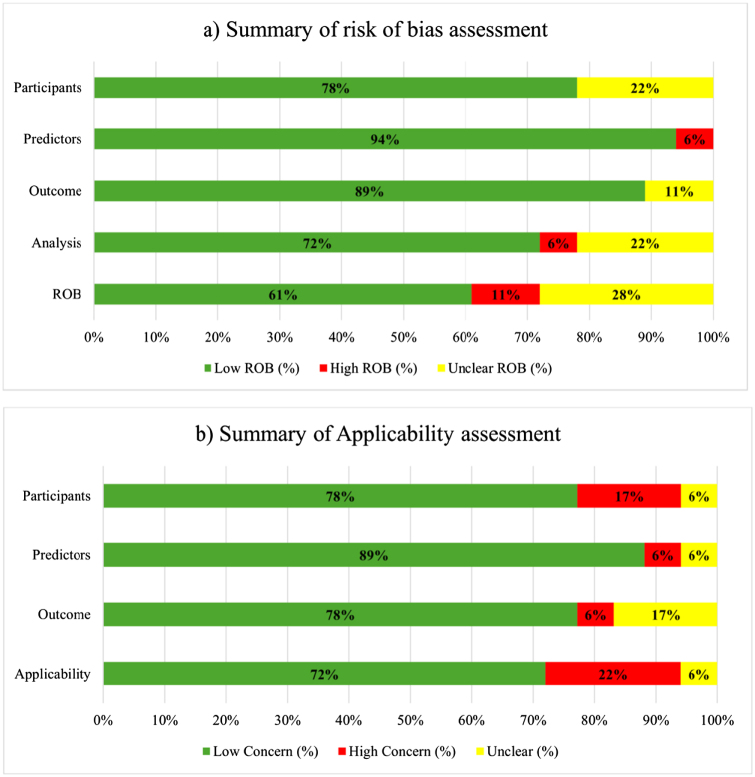
Risk of bias (ROB) and applicability assessment of the included studies.

The ROB and applicability assessments are shown in Table 2 and Figure 2.

**Table 2:** Risk of bias and applicability assessment

Author, Year	Risk of Bias				Applicability			Overall	
	1. Participants	2. Predictors	3. Outcome	4. Analysis	1. Participants	2. Predictors	3. Outcome	Risk of Bias	Applicability
Adams 2013[17]	**+**	**+**	**+**	**+**	**+**	**+**	**+**	**+**	**+**
Francis 2015[18]	**?**	**+**	**+**	-	**?**	**+**	**?**	-	**?**
Chen 2018[19]	**+**	**+**	**+**	**+**	**+**	**+**	**+**	**+**	**+**
Azimi 2019[20]	**?**	**+**	**+**	**+**	-	**+**	**?**	**?**	-
Bosch 2021[21]	**+**	**+**	**?**	**+**	**+**	**+**	**+**	**?**	**+**
Degett 2021[22]	**+**	**+**	**+**	**?**	**+**	**+**	**+**	**?**	**+**
Mazaki 2021[23]	**+**	-	**+**	**?**	**+**	-	**?**	-	-
Wen 2021[24]	**+**	**+**	**+**	**+**	**+**	**+**	**+**	**+**	**+**
Bräuner 2022[25]	**+**	**+**	**+**	**+**	**+**	**+**	**+**	**+**	**+**
Lin 2022[26]	**+**	**+**	**+**	**+**	**+**	**+**	**+**	**+**	**+**
Chen 2023[27]	**+**	**+**	**+**	**+**	**+**	**+**	**+**	**+**	**+**
Shen 2023[28]	**+**	**+**	**+**	**+**	**+**	**+**	**+**	**+**	**+**
Agnes 2024[29]	**+**	**+**	**+**	**+**	**+**	**+**	**+**	**+**	**+**
Brydges 2024[30]	**?**	**+**	**?**	**?**	-	**?**	-	**?**	-
Li 2024[31]	**?**	**+**	**+**	**?**	-	**+**	**+**	**?**	-
Nwaiwu 2024[32]	**+**	**+**	**+**	**+**	**+**	**+**	**+**	**+**	**+**
Taha-Mehlitz 2024[33]	**+**	**+**	**+**	**+**	**+**	**+**	**+**	**+**	**+**
Traeger 2024[34]	**+**	**+**	**+**	**+**	**+**	**+**	**+**	**+**	**+**

Green = low risk of bias; Red = high risk of bias; Yellow = unclear risk of bias.

### Anastomotic leak

**Figure 3.**
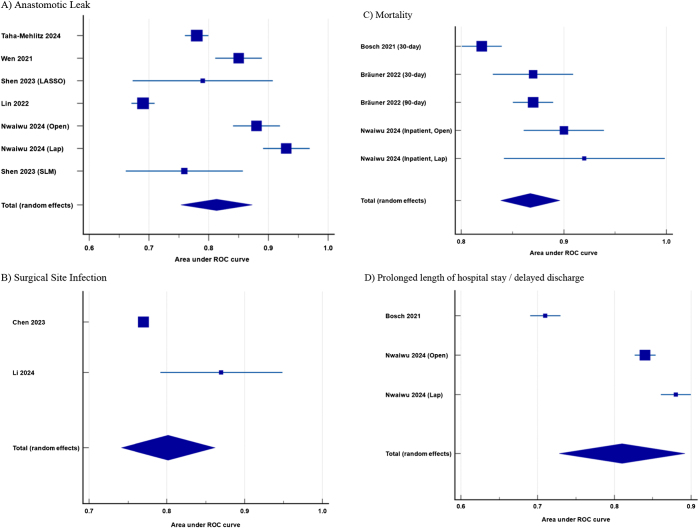
Forest plots of the AUC for (a) anastomotic leak; (b) surgical site infection; (c) mortality; and (d) prolonged length of hospital stay. ROC, receiver operator curve.

Eight studies^[17,23,24,26,28,29,32,33]^ used predictive modeling to assess AL developing postcolorectal surgery, giving a pooled AUC of 0.813 [SE: 0.031, 95% CI (0.753–0.873)] (Fig. 3a). The AUC and accuracy varied by study design and the number of patients included. For example, artificial neural networks (ANNs) achieved the highest AUC of 0.93 in a large study of 14,935 patients[32], while smaller cohorts[33] reported lower AUCs (0.78–0.89).

Key predictors consistently linked to the risk of developing AL were as follows: tumor location in relation to the anal verge (e.g. distances <5 cm), intra-operative factors such as blood loss >100 mL, and postoperative laboratory results [elevated creatinine, C-reactive protein (CRP)].

### Surgical site infection

Postoperative infection was reported in three studies^[20,27,31]^ with two specifically focusing on SSI occurrence^[27,31]^. Both studies used several risk prediction tools, with the ANN models outperforming others. The pooled AUC of the best-performing models was 0.802 [SE: 0.031, 95% CI (0.742–0.862)] (Fig. 3b). For SSIs, surgical approach (open vs laparoscopic), type of suture used for closure, operative time, and preoperative albumin were significant predictive factors.

### Paralytic ileus

Paralytic ileus was assessed in two studies^[30,34]^ with seven different prediction models. These models demonstrated a moderate to strong performance (AUC: 0.80–0.84). Brydges et al[30]. highlighted age, body mass index (BMI), and pre-existing renal dysfunction as critical predictors for the development of paralytic ileus. Traeger et al[34] identified sarcopenia, postoperative hypokalemia, and opioid use as key drivers for this outcome occurring postoperatively.

### Mortality and prolonged length of stay

Four studies^[21,22,25,32]^ reported mortality prediction with a pooled AUC of 0.802 [SE: 0.031, 95% CI (0.742–0.862)] (Fig. 3c). A Danish study[25] of 65,612 patients using LASSO (Least Absolute Shrinkage and Selection Operator) regression achieved an AUC of 0.87, driven by factors like emergency surgery, age, and ASA scores. Similarly, a Dutch analysis[21] of 62,501 patients linked pre-existing co-morbidities such as chronic obstructive pulmonary disease and hypertension to mortality risk.

Prediction for prolonged LOS was reported in two studies^[18,32]^, and the pooled AUC was 0.810 [SE: 0.042, 95% CI (0.728–0.892)] (Fig. 3d).

### Re-admission and bleeding

Re-admission risk was evaluated in three studies^[18,21,29]^. One study[29] reported an AUC of 0.9, with the remaining two achieving much lower values of 0.63[18] and 0.68[21], respectively. Chen et al[19] used gradient boosting and logistic regression models, showing an AUC of 0.81 and 0.77 for postoperative bleeding risk, respectively. The presence of anemia, hemophilia, and nutritional status were the variables most strongly associated with hemorrhage risk in the first postoperative week.

## Discussion

To our knowledge, this is the first systematic review and meta-analysis comprehensively assessing the predictive performance of machine learning models across multiple postoperative complications following colorectal surgery. Eighteen studies^[17–34]^ published between 2013 and 2024, originating from a wide geographical area were included. Analyzed outcomes included AL, postoperative infection, paralytic ileus occurrence, mortality, delayed discharge/increased LOS, re-admissions, and postoperative hemorrhage. Unlike previous narrative reviews, this study quantifies a model’s performance using AI, providing a benchmark for future machine learning studies.

The ability to learn and adapt (without following explicit instructions) using algorithms and statistical modeling, drawing inferences based on pattern recognition, often from large datasets, means that machine learning has potential applications and utility in many fields. Computer-aided diagnosis can assist in many imaging and diagnostic technologies, including X-ray[35], ultrasound[36], magnetic resonance imaging[37], and endoscopy (polyp detection)[38]. In radiology, machine learning algorithms are already employed to detect tumors and predict biomarkers[39]. This technology could be further extrapolated into other fields, including colorectal surgery, using non-linear predictive models to aid clinicians in assessing the likelihood of developing complications following major bowel resections. The use of predictive modeling could, therefore, contribute significantly to improving surgical outcomes and aiding in perioperative decision-making. This will be of great value to both clinicians and patients alike.

The present study highlights the promising potential of machine learning in predicting several outcomes. Eight studies used machine learning to predict AL with a pooled AUC of 0.81. Clinically significant AL remains one of the most severe and dreaded complications following colorectal surgery[40]. It is associated with considerable morbidity and mortality[41] and prolonged LOS[42] and consequently has implications on health care systems for both capacity and costs.

The etiology of AL is both complex and multifactorial[43], often involving both patient and technical factors, including (but not limited to) male gender^[44,45]^, obesity, comorbidities, nutritional status, intra-operative contamination, preoperative treatments (steroids, radiotherapy), and emergency resections[46]. The outcome following such a catastrophic event can be detrimental both oncologically and often, leading to the creation of an ostomy. Therefore, understanding the complex interplay between various risk factors through machine learning that may predispose patients to a higher risk for developing AL will allow more tailored and personalized treatment to mitigate this risk.

For predicting post colorectal surgery mortality using machine learning methods, we calculated a pooled AUC of 0.87. This compared favorably with the UK National Emergency Laparotomy Audit (NELA) risk score (AUC 0.75–0.79)[47]. The NELA risk calculator is a tool used to assess predicted 30-day mortality following an emergency laparotomy. High-risk emergency laparotomy is defined as any patient with a NELA score of ≥ 5%. Other scoring systems quantifying mortality risk following an emergency laparotomy include the American College of Surgeons National Surgical Quality Improvement Score (NSQIP) and the Portsmouth Physiological and Operative Severity Score (P-POSSUM)[48].

The original Physiological and Operative Severity Score for the Enumeration of Mortality and Morbidity (POSSUM)[10], proposed in the early nineties, assesses expected morbidity and mortality based on a physiological score (12 factors) and a surgical severity score (six operative factors). To better predict mortality, the refined P-POSSUM was later introduced[49], collecting the same physiological/operative parameters but using different calculations to determine estimated mortality risk. These scoring systems are utilized to guide better expenditure of finite health care resources in the care of postoperative patients.

We estimated the AUC for these widely used scoring systems and found that machine learning methods were comparatively superior in assessing predicted mortality: AUC values of 0.62 and 0.76 for POSSUM and P-POSSUM, respectively[48]. Both of these established scoring systems have been validated for a number of surgical specialities. However, machine learning techniques may, in the near future, provide a more accurate reflection of the risk faced by patients undergoing increasingly complex gastrointestinal surgery.

The present study also revealed the potential clinical value of machine learning techniques in assessing other outcomes, including predicting prolonged LOS (pooled AUC 0.81) and SSI (AUC 0.80). These are clinically important and relevant findings, particularly in the context of modern health care systems. An ageing population, increasing demands in accessing care, major reforms required in social care, and financial constraints mean that unexpected delays in hospital discharge or deviation from anticipated recovery time can lead to adverse consequences. Delayed discharges have knock-on effects on institutional capacity and a bearing on treatment waiting times.

Therefore, continued innovations and the correct application of validated available technology, including machine learning, are important adjuncts in planning and providing modern health care. They serve as useful guides in helping clinicians with peri-operative decision-making, tailoring and delivering personalized patient care.

Although the aforementioned existing models and scoring systems have been instrumental in clinical decision-making and have been proposed as surgical quality measures, some studies have questioned their validity. For instance, an analysis of colorectal cases assessing the NSQIP risk calculator found it underestimated SSI and overall complication rates[50]. Novel machine learning algorithms may prove useful in preoperative patient education, helping them to understand the rationale for proposed treatment and improving the consent process. For clinicians, better decision-making, including the need for higher levels of postoperative care and interventions to improve surgical outcomes may be achieved through these models[51].

Advances in the field of deep learning, a subset of machine learning, have allowed neural networks to exceed previous approaches to performance. Machine learning aims to find generalizable predictive patterns that differ from statistics (drawing inferences from a sample) with approaches broadly divided into three categories depending on available “feedback” and include supervised, un-supervised, and reinforcement learning[52].

AI-based risk prediction tools have demonstrated their clinical value in a cohort of elderly patients undergoing emergency surgery[53]. The tool predicted in-hospital mortality for patients aged 65 to 85 years with an AUC of 0.80–0.84, acute renal failure (AUC 0.85), respiratory failure (AUC 0.86), and septic shock (AUC 0.90). This allows for improved future decision-making and better patient counseling.

Nwaiwu et al[32] reported three machine learning models predicting AL, prolonged LOS, and inpatient mortality, with high AUC in patients undergoing laparoscopic and open surgery for colorectal malignancy. These results compared favorably with other studies using machine learning techniques to predict disease recurrence and risk of developing colorectal cancer. Merath et al[7] used machine learning to predict postoperative complications after liver, pancreatic, and colorectal surgery. Decision tree models were used, and the authors reported good results in predicting specific complications, outperforming established methods such as the ASA and NSQIP risk calculator.

In modern surgical practice, especially for cancer treatment and using a multi-disciplinary team approach, validated machine learning models can be integrated into existing patient pathways. The benefits of this are early recognition of the high-risk surgical patient and initiation of interventions to mitigate identified risks. For instance, such patients may be suitable for preoperative rehabilitation and optimization, or following risk assessment, it may be decided via consensus to pursue non-surgical treatment. Alternatively, with robust, highly accurate modeling systems, clinicians may decide preoperatively on the creation of a temporary/permanent stoma and communicate these decisions and counsel patients appropriately. Moreover, risk-prediction models may suggest the need for higher levels of care postoperatively and these too can be planned well in advance. The advantages of machine learning algorithms over established risk predictors are a non-linear approach, use of large, complex datasets to identify patterns, and provide flexibility[34].

Several machine learning techniques were used in the included studies, including ANNs or connectionist systems based on nodes called “artificial neurons,” decision tree classifier, support-vector networks, random forest (RF), and many more[52]. Each has its own strengths and advantages. For instance, decision trees are easy to interpret, similar to a flowchart showing how decisions are reached[54]. Logistic regression, meanwhile, highlights factors strongly influencing outcomes and provides more clear numerical insights.

Overfitting is a common challenge with these models and occurs when a tool works perfectly on the data it is trained on, but struggles with new real-world patient data. To address this, researchers often combine multiple models, balancing accuracy with reliability, as combining methods reduces the risk of introducing errors[27].

While the included articles introduced a wide range of features, some studies[28] used only 6–10 features and others[26] used 58–111 features. Attempting to define relevant features is critical in predicting the dependent variable. Excluding redundant and irrelevant features during modeling will help improve overall performance[20].

In this review, some of the included studies used patient records to train machine learning using extensive datasets^[27,32]^, whilst others[17] used smaller cohorts. Datasets require delicate handling of missing values, outlier detection, normalization, encoding categorical variables, and feature engineering. Imbalanced data requires additional processing, re-sampling, and class weighting to optimize model performance.

This study is not without its limitations. Firstly, regarding model preferences, ANNs and random forests were favored for high-dimensional data, while LASSO and logistic regression provided interpretability for smaller datasets. Secondly, data challenges were encountered, and studies with less than 500 patients often reported inflated AUCs (AUC: 0.89, 76 patients)[17], leading to concerns of overfitting. Conversely, large registry-based studies[27] prioritized generalizability but faced missing data issues. A further limitation concerns the pooling of AUC values from diverse machine learning models. Given the heterogeneity in algorithms, model architecture, input variables, training data, validation methods, and population characteristics, such pooling may not be methodologically robust. While pooling AUCs provides a general impression of a given model’s performance, it oversimplifies the inherent complexity associated with AI models. Future work should consider subgroup analyses or more refined approaches such as meta-regression to account for and factor this variability[55].

## Conclusions

Machine learning algorithms and methods have promising clinical utility and applicability in accurately predicting patient risk of developing complications following colorectal surgery. These tools demonstrate superiority over presently available linear risk prediction calculators used by clinicians to aid prognostication; however, machine learning tools require rigorous validation before replacing established risk calculators. Their current use should be as an adjunct while further validation studies emerge. Future well-designed studies are needed to fully appreciate and understand the power of machine learning in various medical settings, including surgery.

## Data Availability

All data relevant to this study are included in the article. Additional data are available on request from the corresponding author.

## Research Registration Unique Identifying Number (UIN)

CRD42025638998.

## References

[1] Morgan E Arnold M Gini A. Global burden of colorectal cancer in 2020 and 2040: incidence and mortality estimates from GLOBOCAN. Gut 2023;72:338–44. 36604116 10.1136/gutjnl-2022-327736

[2] World Health Organization. Colorectal cancer. Accessed February 2025. https://www.who.int/news-room/fact-sheets/detail/colorectal-cancer

[3] Warps AK Tollenaar RAEM Tanis PJ Dekker JWT. Dutch ColoRectal Audit. Postoperative complications after colorectal cancer surgery and the association with long-term survival. Eur J Surg Oncol 2022;48:873–82. 34801319 10.1016/j.ejso.2021.10.035

[4] Chiarello MM Fransvea P Cariati M Adams NJ Bianchi V Brisinda G. Anastomotic leakage in colorectal cancer surgery. Surg Oncol 2022;40:101708. 35092916 10.1016/j.suronc.2022.101708

[5] Rennie O Sharma M Helwa N. Colorectal anastomotic leakage: a narrative review of definitions, grading systems, and consequences of leaks. Front Surg 2024;11:1371567. 38756356 10.3389/fsurg.2024.1371567 PMC11097957

[6] Kufel J Bargieł-Łączek K Kocot S. What is machine learning, Artificial Neural Networks and Deep Learning? Examples of practical applications in medicine. Diagnostics (Basel) 2023;13:2582. 37568945 10.3390/diagnostics13152582 PMC10417718

[7] Merath K Hyer JM Mehta R. Use of machine learning for prediction of patient risk of postoperative complications after liver, pancreatic, and colorectal surgery. J Gastrointest Surg 2020;24:1843–51. 31385172 10.1007/s11605-019-04338-2

[8] National Guideline Centre (UK). Evidence review for preoperative risk stratification tools: perioperative care in adults: evidence review C. London: National Institute for Health and Care Excellence (NICE); 2020. (NICE Guideline, No. 180). https://www.ncbi.nlm.nih.gov/books/NBK561975/32931173

[9] Chudgar N Yan S Hsu M. The American College of Surgeons Surgical Risk Calculator performs well for pulmonary resection: a validation study. J Thorac Cardiovasc Surg 2022;163:1509–1516.e1. 33610360 10.1016/j.jtcvs.2021.01.036 PMC8292429

[10] Copeland GP Jones D Walters M. POSSUM: a scoring system for surgical audit. Br J Surg 1991;78:355–60. 2021856 10.1002/bjs.1800780327

[11] Higgins JPT Green S Chandler J. eds. Cochrane Handbook for Systematic Reviews of Interventions version 6.5. Cochrane; 2011. Updated August 2024. www.cochrane.org/handbook

[12] PRISMA Group, Moher D Liberati A Tetzlaff J Altman DG. Preferred reporting items for systematic reviews and metaanalyses: the PRISMA statement. Int J Surg 2010;8:336–41. 20171303 10.1016/j.ijsu.2010.02.007

[13] Shea BJ , Reeves BC , Wells G. AMSTAR 2: a critical appraisal tool for systematic reviews that include randomised or non-randomised studies of healthcare interventions, or both. BMJ 2017;358:j4008. 28935701 10.1136/bmj.j4008 PMC5833365

[14] Agha RA Mathew G Rashid R. Transparency In The reporting of Artificial INtelligence– the TITAN guideline. Prem J Sci 2025;10:100082.

[15] Wolff RF Moons KGM Riley RD *et al.* PROBAST Group†. PROBAST: a tool to assess the risk of bias and applicability of prediction model studies. Ann Intern Med 2019;170:51–58. 30596875 10.7326/M18-1376

[16] Higgins JPT , Li T , Deeks JJ, eds. Chapter 6: Choosing effect measures and computing estimates of effect [last updated August 2023]. In: Higgins JPT, Thomas J, Chandler J. eds. Cochrane Handbook for Systematic Reviews of Interventions version 6.5. Cochrane; 2024. cochrane.org/handbook.

[17] Adams K Papagrigoriadis S. Creation of an effective colorectal anastomotic leak early detection tool using an artificial neural network. Int J Colorectal Dis 2014;29:437–43. 24337715 10.1007/s00384-013-1812-8

[18] Francis NK Luther A Salib E. The use of artificial neural networks to predict delayed discharge and readmission in enhanced recovery following laparoscopic colorectal cancer surgery. Tech Coloproctol 2015;19:419–28. 26084884 10.1007/s10151-015-1319-0

[19] Chen D Afzal N Sohn S. Postoperative bleeding risk prediction for patients undergoing colorectal surgery. Surgery 2018;164:1209–16. 30033185 10.1016/j.surg.2018.05.043 PMC6263850

[20] Azimi K Honaker MD Chalil Madathil S Khasawneh MT. Post-operative infection prediction and risk factor analysis in colorectal surgery using data mining techniques: a pilot study. Surg Infect (Larchmt) 2020;21:784–92. 32155386 10.1089/sur.2019.138

[21] van den Bosch T Warps AK de Nerée Tot Babberich MPM. Dutch ColoRectal Audit. Predictors of 30-Day Mortality Among Dutch Patients Undergoing Colorectal Cancer Surgery, 2011-2016. JAMA Netw Open 2021;4:e217737. Erratum in: JAMA Netw Open 2021;4(8):e2127694. doi:10.1001/jamanetworkopen.2021.27694 33900400 10.1001/jamanetworkopen.2021.7737 PMC8076964

[22] Degett TH Christensen J Dalton SO. Prediction of the postoperative 90-day mortality after acute colorectal cancer surgery: development and temporal validation of the ACORCA model. Int J Colorectal Dis 2021;36:1873–83. 33982139 10.1007/s00384-021-03950-6

[23] Mazaki J Katsumata K Ohno Y. A novel predictive model for anastomotic leakage in colorectal cancer using Auto-artificial Intelligence. Anticancer Res 2021;41:5821–25. 34732457 10.21873/anticanres.15400

[24] Wen R Zheng K Zhang Q. Machine learning-based random forest predicts anastomotic leakage after anterior resection for rectal cancer. J Gastrointest Oncol 2021;12:921–32. 34295545 10.21037/jgo-20-436 PMC8261311

[25] Bräuner KB Rosen AW Tsouchnika A. Developing prediction models for short-term mortality after surgery for colorectal cancer using a Danish national quality assurance database. Int J Colorectal Dis 2022;37:1835–43. 35849195 10.1007/s00384-022-04207-6

[26] Lin V Tsouchnika A Allakhverdiiev E. Training prediction models for individual risk assessment of postoperative complications after surgery for colorectal cancer. Tech Coloproctol 2022;26:665–75. 35593971 10.1007/s10151-022-02624-x

[27] Chen KA Joisa CU Stem JM Guillem JG Gomez SM Kapadia MR. Improved prediction of surgical-site infection after colorectal surgery using machine learning. Dis Colon Rectum 2023;66:458–66. 36538699 10.1097/DCR.0000000000002559 PMC10069984

[28] Shen Y Huang LB Lu A Yang T Chen HN Wang Z. Prediction of symptomatic anastomotic leak after rectal cancer surgery: a machine learning approach. J Surg Oncol 2024;129:264–72. 37795583 10.1002/jso.27470

[29] Agnes A Nguyen ST Konishi T. Early postoperative prediction of complications and readmission after colorectal cancer surgery using an Artificial Neural Network. Dis Colon Rectum 2024;67:1341–52. 38959458 10.1097/DCR.0000000000003253

[30] Brydges G Chang GJ Gan TJ Konishi T Gottumukkala V Uppal A. Testing machine learning models to predict postoperative ileus after colorectal surgery. Curr Oncol 2024;31:3563–78. 38920745 10.3390/curroncol31060262 PMC11202731

[31] Li J Yan Z. Machine learning model predicting factors for incisional infection following right hemicolectomy for colon cancer. BMC Surg 2024;24:279. 39354475 10.1186/s12893-024-02543-8 PMC11443797

[32] Nwaiwu CA Rivera Perla KM Abel LB. Predicting Colonic Neoplasia Surgical complications: a machine learning approach. Dis Colon Rectum 2024;67:700–13. 38319746 10.1097/DCR.0000000000003166

[33] Taha-Mehlitz S Wentzler L Angehrn F. Machine learning-based preoperative analytics for the prediction of anastomotic leakage in colorectal surgery: a swiss pilot study. Surg Endosc 2024;38:3672–83. 38777894 10.1007/s00464-024-10926-4 PMC11219450

[34] Traeger L Bedrikovetski S Hanna JE Moore JW Sammour T. Machine learning prediction model for postoperative ileus following colorectal surgery. ANZ J Surg 2024;94:1292–98. 38695239 10.1111/ans.19020

[35] Ahmad HK Milne MR Buchlak QD. Machine Learning Augmented Interpretation of Chest X-rays: a Systematic Review. Diagnostics (Basel) 2023;13:743. 36832231 10.3390/diagnostics13040743 PMC9955112

[36] Kim YH. Artificial intelligence in medical ultrasonography: driving on an unpaved road. Ultrasonography 2021;40:313–17. 34053212 10.14366/usg.21031 PMC8217795

[37] Gassenmaier S Küstner T Nickel D. Deep learning applications in magnetic resonance imaging: has the future become present? Diagnostics (Basel) 2021;11:2181. 34943418 10.3390/diagnostics11122181 PMC8700442

[38] van der Sommen F de Groof J Struyvenberg M. Machine learning in GI endoscopy: practical guidance in how to interpret a novel field. Gut 2020;69:2035–45. 32393540 10.1136/gutjnl-2019-320466 PMC7569393

[39] Bektaş M Tuynman JB Costa Pereira J Burchell GL van der Peet DL. Machine learning algorithms for predicting surgical outcomes after colorectal surgery: a systematic review. World J Surg 2022;46:3100–10. 36109367 10.1007/s00268-022-06728-1 PMC9636121

[40] Tsalikidis C Mitsala A Mentonis VI. Predictive factors for anastomotic leakage following colorectal cancer surgery: where are we and where are we going? Curr Oncol 2023;30:3111–37. 36975449 10.3390/curroncol30030236 PMC10047700

[41] Tonini V Zanni M. Impact of anastomotic leakage on long-term prognosis after colorectal cancer surgery. World J Gastrointest Surg 2023;15:745–56. 37342854 10.4240/wjgs.v15.i5.745 PMC10277951

[42] Tsai YY Chen WT. Management of anastomotic leakage after rectal surgery: a review article. J Gastrointest Oncol 2019;10:1229–37. 31949944 10.21037/jgo.2019.07.07 PMC6955017

[43] Alverdy JC Schardey HM. Anastomotic leak: toward an understanding of its root causes. J Gastrointest Surg 2021;25:2966–75. 34100248 10.1007/s11605-021-05048-4 PMC8815793

[44] Aliyev V Tokmak H Goksel S. Robotic Sphincter-saving total mesorectal excision for Rectal Cancer Treatment: a Single-Surgeon Experience in 103 consecutive male patients. Surg Technol Int 2020;37:93–98. 32634247

[45] Aliyev V Piozzi GN Shadmanov N. Robotic and laparoscopic sphincter-saving resections have similar peri-operative, oncological and functional outcomes in female patients with rectal cancer. Updates Surg 2023;75:2201–09. 37955804 10.1007/s13304-023-01686-2

[46] Zarnescu EC Zarnescu NO Costea R. Updates of risk factors for anastomotic leakage after colorectal surgery. Diagnostics (Basel) 2021;11:2382. 34943616 10.3390/diagnostics11122382 PMC8700187

[47] Barghash M Iskandar A Fawzy SI. Predictive performance of NELA versus P-POSSUM mortality scores: are we underestimating the risk of mortality following emergency laparotomy? Cureus 2022;14:e32859. 36694527 10.7759/cureus.32859 PMC9867845

[48] Hansted AK Storm N Burcharth J. Validation of the NELA risk prediction model in emergency abdominal surgery. Acta Anaesthesiol Scand 2023;67:1194–201. 37353882 10.1111/aas.14294

[49] Prytherch DR Whiteley MS Higgins B Weaver PC Prout WG Powell SJ. POSSUM and Portsmouth POSSUM for predicting mortality. Physiological and Operative Severity Score for the enUmeration of Mortality and morbidity. Br J Surg 1998;85:1217–20. 9752863 10.1046/j.1365-2168.1998.00840.x

[50] Adegboyega TO Borgert AJ Lambert PJ Jarman BT. Applying the National Surgical Quality Improvement Program risk calculator to patients undergoing colorectal surgery: theory vs reality. Am J Surg 2017;213:30–35. 27424043 10.1016/j.amjsurg.2016.04.011

[51] Loftus TJ Tighe PJ Filiberto AC. Artificial Intelligence and surgical decision-making. JAMA Surg 2020;155:148–58. 31825465 10.1001/jamasurg.2019.4917 PMC7286802

[52] Woodman RJ Mangoni AA. A comprehensive review of machine learning algorithms and their application in geriatric medicine: present and future. Aging Clin Exp Res 2023;35:2363–97. 37682491 10.1007/s40520-023-02552-2 PMC10627901

[53] Maurer LR Chetlur P Zhuo D. Validation of the Al-based Predictive OpTimal Trees in Emergency Surgery Risk (POTTER) Calculator in Patients 65 Years and older. Ann Surg 2023;277:e8–e15. 33378309 10.1097/SLA.0000000000004714

[54] Blockeel H Devos L Frénay B Nanfack G Nijssen S. Decision trees: from efficient prediction to responsible AI. Front Artif Intell 2023;6:1124553. 37565044 10.3389/frai.2023.1124553 PMC10411911

[55] Van Calster B McLernon DJ van Smeden M Wynants L Steyerberg EW, Topic Group ‘Evaluating diagnostic tests and prediction models’ of the STRATOS initiative. Calibration: the Achilles heel of predictive analytics. BMC Med 2019;17:230. 31842878 10.1186/s12916-019-1466-7 PMC6912996

